# TNAP as a New Player in Chronic Inflammatory Conditions and Metabolism

**DOI:** 10.3390/ijms22020919

**Published:** 2021-01-18

**Authors:** Stephanie Graser, Daniel Liedtke, Franz Jakob

**Affiliations:** 1 Bernhard-Heine-Center for Locomotion Research, Department of Orthopedics, Julius-Maximilians-University Würzburg, 97076 Würzburg, Germany; f-jakob.klh@uni-wuerzburg.de; 2 Institute for Human Genetics, Biocenter, Julius-Maximilians-University Würzburg, 97074 Würzburg, Germany; liedtke@biozentrum.uni-wuerzburg.de

**Keywords:** TNAP, Hypophosphatasia, HPP, mineralization, nervous system, inflammation

## Abstract

This review summarizes important information on the ectoenzyme tissue-nonspecific alkaline phosphatase (TNAP) and gives a brief insight into the symptoms, diagnostics, and treatment of the rare disease Hypophosphatasia (HPP), which is resulting from mutations in the TNAP encoding *ALPL* gene. We emphasize the role of TNAP beyond its well-known contribution to mineralization processes. Therefore, above all, the impact of the enzyme on central molecular processes in the nervous system and on inflammation is presented here.

## 1. Structure, Function, and Substrates of TNAP

Tissue-nonspecific alkaline phosphatase (TNAP) or liver/bone/kidney alkaline phosphatase is an ectoenzyme that is anchored to the outer cell membrane (e.g. in osteoblasts) and to extracellular vesicles via its glycosyl-inositol-phosphate (GPI)-anchor [1,2]. TNAP belongs to the family of alkaline phosphatases (AP) that comprises in humans three additional tissue-specific isoforms: placental (PLAP, *ALPP* National Center for Biotechnology Information NCBI GeneID: 250), germ cell (GCAP, *ALPG* NCBI GeneID: 251), and intestinal alkaline phosphatase (IAP, *ALPI* NCBI GeneID: 248) [2,3].

As an ectophosphatase, TNAP catalyzes the dephosphorylation of its main substrates pyrophosphate (PP_i_) and pyridoxal-5′-phosphate (PLP) in the extracellular space [1,4,5]. In addition to the prominent mineralization inhibitor PP_i_, TNAP further dephosphorylates phosphorylated osteopontin (p-OPN, encoded by the *SSP1* gene), which is preventing hydroxyapatite (HA) crystallization in its phosphorylated state [6]. Moreover, TNAP is also capable of dephosphorylating microbial products, such as lipopolysaccharides (LPS) as well as other phosphorylated small-molecule Toll-like-receptor (TLR) ligands [7]. Beyond that, TNAP is an ectonucleotidase that degrades adenosine triphosphate (ATP) in a stepwise manner to adenosine, with adenosine diphosphate (ADP) and adenosine monophosphate (AMP) as intermediates [1,8]. Consequently, TNAP’s enzymatic function is considerably influencing purinergic signaling cascades as it either provides or decreases the prevalence of substrates for several receptors (P1, P2X, P2Y).

Extrapolating from a structural analysis of the placental AP, a model for the protein structure of TNAP was developed, which implies that the enzyme contains two Zn^2+^ ions and one Mg^2+^ ion in its active center [9]. The function of an additionally identified Ca^2+^ site is still unclear [10]. The molecular weight of TNAP is approximately 80 kDa in its mature, post-translationally (above all, *N*-glycosylated) modified state [11,12]. Distinct protein domains have been characterized, namely the N-terminal α-helix, the crown domain, and the metal binding domain [9]. TNAP monomers form dimers via hydrophobic regions and the enzyme is mostly active as a GPI-anchored homodimer [13]. Altered dimerization can result in dominant negative effects of heterozygous mutations in the *ALPL* gene, which has already been described in the context of Hypophosphatasia (HPP) patients [14,15,16]. This implies that a deficient TNAP monomer is able to decrease the overall activity of its actually fully functional binding partner, either due to steric changes caused by dimerization or due to protein transport failure to the membrane [14,15,17].

The human *ALPL* gene (NCBI GeneID: 249) is located on chromosome 1 (1p36), contains 12 exons, including 11 coding exons as well as two alternative variants of exon 1 (1B and 1L, bone and liver variant), and has an overall gene size of approximately 50 kb [11,18]. Genetic variants resulting in disease-causing mutations have been described extensively [14,16,19,20,21,22] and to a certain extent, their molecular consequences can be predicted by bioinformatic tools [23]. As expected, mutations in essential functional regions that, for example, are coding for the active center or the dimerization domain, are predicted to result in more severe symptoms than mutations in sequences coding for less essential parts of the TNAP protein [23]. Nevertheless, it is important to mention that a definitive correlation between a certain genotype and a resulting phenotype (genotype–phenotype correlation) cannot be predicted and that additional modifying factors can strongly influence the disease manifestation [24]. One example was reported in a family with two affected daughters with the same *ALPL* mutation, where one girl developed a severe and the other a mild HPP onset [22]. These variable genotype–phenotype correlations pose difficulties for genetic counseling and an optimized disease management in HPP [25]. The majority of reported mutations in the human *ALPL* gene are missense mutations that are resulting in amino acid changes (https://www.ncbi.nlm.nih.gov/clinvar/?term=alpl%5Bgene%5D, accessed on 11 December 2020).

## 2. Heritable TNAP Dysfunction: The Rare Disease Hypophosphatasia

### 2.1. General Information on HPP

Genetically inheritable TNAP dysfunction, due to mutations in the *ALPL* gene, leads to the rare disease Hypophosphatasia (HPP) [1,26,27]. The prevalence of HPP is 1:300,000 for severe and approximately 1:6000 for mild courses of disease in the European population [28]. Rarely occurring severe manifestations of HPP are inherited in an autosomal recessive manner. Compound heterozygosity, implying that one patient carries two different mutations in the *ALPL* gene in a heterozygous manner, is very common in HPP patients and results in disease manifestations of very variable severity [14,16,29]. Mild to moderate forms follow an autosomal dominant or recessive trait [16]. Interestingly, all HPP patients displaying mild forms of HPP show a high variety of different genetic variants, including missense, splice site, frameshift, and nonsense mutations [16]. Mornet et al. only recently showed that dominant negative effects are resulting from missense mutations, which lead to moderate HPP forms, whereas haploinsufficiency is considered as the main cause for mild adult forms [16].

### 2.2. Clinical Subtypes

In general, HPP can lead to very heterogeneous outcomes and the patients can be categorized in six clinical subtypes, based on the age at diagnosis and/or of onset [1,30,31,32]. There are mild clinical subtypes, like Perinatal benign, Adult, or Odonto-HPP, with the latter only affecting the mineralization and the anchorage of teeth and leading to premature loss of deciduous teeth as the most prominent symptom [1,27,31,32,33,34,35]. Severe forms of HPP, like the Perinatal lethal or the Infantile subtypes, can both lead to a premature death [1,30,31,32]. Perinatal lethal forms often go along with epileptic seizures and respiratory failure, whereas Infantile HPP may go along with premature closure of the cranial sutures of the skull (craniosynostosis) [30,31,32]. The sixth described clinical subtype is Childhood HPP, which leads to symptoms like limb shortening, delayed walking, a characteristic waddling gait, and hypo-mineralization, and is not considered as life-threatening [27,30].

### 2.3. Diagnosis

Generally, the most prominent symptoms of HPP are hypo-mineralization of bones and teeth [1,26,31,36,37,38,39]. Nevertheless, lungs, kidneys, and the nervous system can also be affected, depending on the severity of the disease [21,27,31,36]. Additionally, chronic pain is an issue that goes along with HPP and that significantly reduces the patients’ quality of life [40]. Therefore, HPP is considered a multisystemic disease and should optimally be treated by an interdisciplinary team of physicians [32,40,41]. The diagnosis of HPP includes observance of characteristic symptoms, measurement of decreased serum AP activity (based on age- and sex-specific reference values), detection of increased PLP concentrations in the blood serum [42], as well as phosphoethanolamine (PEA) in the urine, and the detection of mutations in the *ALPL* gene sequence [43]. Radiologic imaging techniques may be used to further confirm the diagnosis [43,44]. Due to HPP being a rare disease with variable severity levels and symptoms, patients are often misdiagnosed or have to wait for years in order to get the correct diagnosis, which may lead to improper treatment in the meantime and can cause high psychological burden [45].

### 2.4. Treatment

Despite that functional and genetic understanding of HPP has greatly advanced in recent years, no curative treatment for HPP is available to date. Therefore, patients are still treated symptomatically, which includes, among others, treatment with non-steroidal anti-inflammatory drugs (NSAIDs) against inflammation [46,47], neurosurgery due to issues caused by craniosynostosis [48], treatment of dental problems by dentists [34,49], and eventually, orthopedic surgery after the occurrence of fractures [50,51,52]. An enzyme replacement therapy with a recombinant enzyme including a deca-aspartate anchor, called Asfotase alfa, has been approved for treatment of severely affected children since 2015 in Europe and the U.S. (https://www.ema.europa.eu/en/medicines/human/EPAR/strensiq; https://www.accessdata.fda.gov/drugsatfda_docs/nda/2015/125513Orig1s000TOC.cfm, accessed on 14 December 2020) [53,54,55,56]. Several studies have analyzed the pharmacodynamics and safety of Asfotase alfa and additionally shown its positive effects on survival rates, dental health, skeletal development, motor functions, and respiratory functions in mouse models and HPP patients [53,54,55,56,57,58,59,60,61,62]. Furthermore, treatment of HPP patients with a sclerostin antibody and the osteoporosis medication teriparatide have already been described, but these approaches primarily target bone mass and fracture healing rather than substituting or supporting TNAP activity [63,64,65,66,67,68,69].

## 3. TNAP as an Ectophosphatase—The Molecular Role of TNAP in Mineralization Processes

The prominent skeletal and dental phenotype of HPP patients indicates a central role of TNAP within the mineralization process, including the crystallization of HA as a central step. Interestingly, TNAP is an important promoter of the mineralization process as it dephosphorylates central mineralization inhibitors, like PP_i_ and p-OPN [6,70]. Generally, TNAP’s function is to propagate HA crystallization, rather than to initiate this process within the matrix vesicles [71]. In general, HA crystallization is controlled by coordinated activity of enzymes and channels, which regulate the availability of PP_i_ and ATP in the extracellular microenvironment for the production of P_i_ as an essential partner for Ca^2+^ to form HA. Here, among others, ENPP1 (ectonucleotide pyrophosphatase/phosphodiesterase family member 1), ANKH (progressive ankylosis protein homolog), PHOSPHO1 (phosphatase orphan 1), and PANX1 (pannexin 1) are central molecular players [70]. An increase of PP_i_ prevalence in the extracellular space is mediated by its transport from the inside of the cell to the extracellular matrix (ECM) through the channel ANKH and additionally, via the enzymatic activity of ENPP1 [70]. The transmembrane channel PANX1 promotes the transport of ATP to the extracellular microenvironment, where the enzymes ENPP1 and TNAP can subsequently degrade it [70,72]. PHOSPHO1 dephosphorylates PP_i_ to P_i_ inside matrix vesicles in order to promote mineralization, however, in a non-redundant manner to TNAP that is membrane-bound and acting extracellularly [71,73].

## 4. The Molecular Role of TNAP in Neuronal Biology and Neurotransmitter Metabolism

Beyond their often more prominently manifesting mineralization phenotype, HPP patients show neurological symptoms, including epileptic seizures, depression, anxiety, and sleeping disorders (in an increased prevalence compared to the general U.S. population) [74]. Epileptic seizures occur in severely affected children but not in adult patients with milder onset [74,75,76]. Those seizures are often pyridoxine-dependent and have been linked to TNAP´s biochemical function to indirectly provide the brain with PLP, which is an important co-factor for central enzymes of the neurotransmitter metabolism in the central nervous system, e.g., glutamate decarboxylase (GAD) and aromatic L-amino acid decarboxylase (AADC) [75,77,78]. This seems paradoxical at first glance, but HPP patients, who display increased concentrations of PLP within their blood serum, exhibit a lack of the same molecule within the brain, as only the dephosphorylated form (PL), which is provided by TNAP, is able to cross the blood–brain barrier (BBB), and is subsequently re-phosphorylated [75]. Going along with this, TNAP-deficient mouse brains show aberrant concentrations of several neurotransmitters, e.g., gamma-aminobutyric acid (GABA) and adenosine, as well as of molecules that are involved in the myelin synthesis, e.g., *N*-acetyl-aspartate and *N*-acetyl-aspartyl-glutamate [79]. Further, evidence was provided for TNAP’s expression in neuronal cells, for its impact on the PLP-dependent synthesis of serotonin and catecholamines, and for its influence on the dephosphorylation of laminin [80].

Besides neurotransmission, TNAP has an influence on myelination and is exclusively expressed in myelin-free areas of the axon, which has already been described in mice and in primates [81,82]. Furthermore, the enzyme has an impact on the development and the functionality of synapses [81,82,83]. An additional observation is that the length of neurites is influenced by TNAP activity, as the purinergic receptor P2X7, which mediates an inhibitory effect on the axonal outgrowth and cannot be activated when TNAP degrades its ligand ATP to adenosine in a stepwise manner [84,85]. Furthermore, *Akp2*^−/−^ mice (TNAP knockout mice) develop enhanced proliferation of neuronal precursor cells as well as changes concerning morphology and activity of neurons due to a downregulation of P2X7 receptor expression [86]. Interestingly, PLP is an antagonist of the P2X7 receptor, which provides a second option for TNAP to influence the activity of the receptor [87]. Taking a closer look at TNAP’s impact on purinergic signaling, effects on sleeping, epileptic seizures, and the perception of pain become apparent [8,86,88,89]. Both sleeping disorders and pain indeed occur in HPP patients [40,74].

TNAP’s molecular role in stem cells has been controversially discussed in the literature, which can eventually be reconciled by different roles on different levels of commitment. TNAP knockdown increases the proliferation of neuronal precursor cells in mice [86] and decreases the proliferation (and differentiation) of murine neural stem cells in vitro [90]. A case report describes that TNAP deficiency, due to two compound heterozygous null mutations in the *ALPL* gene, can result in severe and progressive encephalopathy with a fatal onset of the disease [21].

## 5. The Molecular Role of TNAP in Inflammation Processes

**Figure 1:**
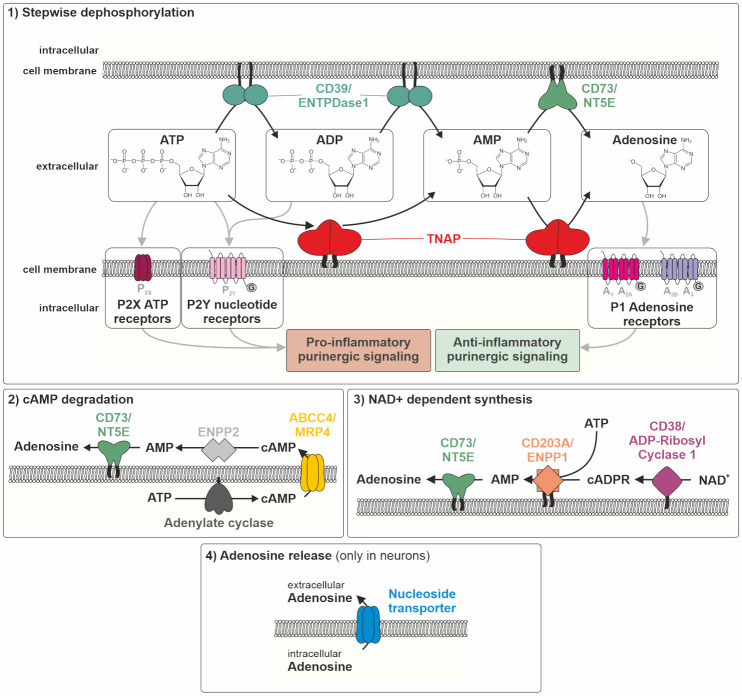
Balance between pro- and anti-inflammatory signaling depending on purine metabolizing enzymes. ABCC4/MRP4: ATP binding cassette subfamily C member 4/multidrug resistance protein 4, NAD^+^: nicotinamide adenine dinucleotide, ATP/ADP/AMP: adenosine tri-/di-/monophosphate, cADPR: cyclic adenosine 5′-diphosphate ribose, cAMP: cyclic adenosine monophosphate, CD39/ENTPDase 1: ectonucleoside triphosphate diphosphohydrolase 1, CD73/NT5E: ecto-5’-nucleotidase, ENPP1/2: ectonucleotide pyrophosphatase/phosphodiesterase 1/2, TNAP: tissue-nonspecific alkaline phosphatase. Note: Adenosine release via nucleoside transporters, depicted in (4), is only prevalent in neurons.

**Figure 2:**
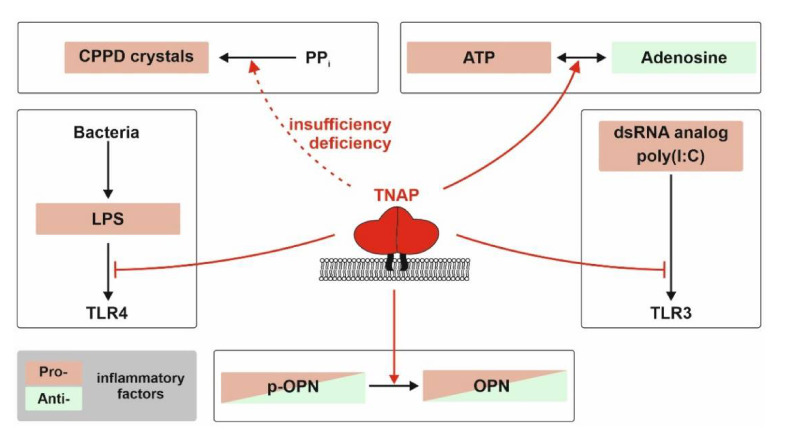
TNAP’s influence on the regulation of inflammation processes. ATP: adenosine triphosphate, CPPD: calcium pyrophosphate dehydrate, dsRNA: double-stranded ribonucleic acid, LPS: lipopolysaccharide, OPN: osteopontin, poly(I:C): poly-inosine:cytosine, p-OPN: phosphorylated osteopontin, PP_i_: inorganic pyrophosphate, TLR3: toll-like receptor 3, TLR4: toll-like receptor 4, TNAP: tissue-nonspecific alkaline phosphatase. Note: All mechanisms depicted are solid in terms of data, except the role of p-OPN vs. OPN on anti-/pro-inflammatory-effects. Green: anti-inflammatory effects, red: pro-inflammatory effects.

TNAP activity has recently been linked to new scientific fields, including inflammation and metabolism. In HPP, as a disease model for TNAP deficiency, inflammatory conditions, such as bone marrow edema/chronic non-bacterial osteomyelitis, myopathies, tendinitis, and increased predisposition for periodontitis, are very common in both children and adults [27,31,36,91]. Provided that TNAP deficiency contributes to inflammatory reactions, e.g., in bone and muscle in HPP patients, research groups from different fields teamed up to unravel the molecular mechanisms of TNAP activity that modulate inflammatory processes. Both the ectophosphatase- and the ectonucleotidase-related functions contribute to the initiation and modulation of inflammation in variable tissues and conditions. Moreover, either accumulation of substrates as well as delivery of products can be involved in pro- and anti-inflammatory scenarios. Especially, TNAP’s role concerning the balance between pro-inflammatory ATP effects and anti-inflammatory effects of its breakdown product adenosine has recently received attention (see Figure 1 and Figure 2); hence, chemical agonists and antagonists have been developed and are currently being studied in clinical trials [92,93,94,95,96,97].

Impaired TNAP ectophosphatase activity causes PP_i_ accumulation, which can initiate the generation of calcium pyrophosphate dehydrate (CPPD) crystals. Crystals accumulate in the joints and tissues of HPP patients and consequently initiate inflammation processes [98]. Those crystals stimulate transmembrane scavenger receptors and/or are phagocytosed, which triggers both the NLRP3 inflammasome and a process called necroinflammation [99]. The latter is a process of inflammation-related regulated necrosis, which can either occur in the phagocytosing cell (e.g., primarily macrophages) or in any neighboring cells via danger-associated molecular pattern (DAMP) signaling that activates the innate immune reaction [99]. Osteopontin, a protein substrate of TNAP, is not only involved in mineralization but also in a broad variety of processes, above all in inflammation, cytokine secretion and consecutive insulin resistance, and even inflammation-related tumorigenesis [100,101,102]. It is generally assigned as a putatively “proinflammatory protein”, however, both pro- and anti-inflammatory attitudes have been described and the role of phosphorylation and dephosphorylation has rarely been addressed in this context (see Figure 2). Recent work describes that recombinant osteopontin mediates anti-inflammatory effects in brain microglia [103]. Other reports render it a candidate orchestrator of chronic inflammatory processes in almost any tissue and organ, among others, in muscle, joints, kidney, and liver [104,105]. More research is definitely needed to dissect whether TNAP-related dephosphorylation balances anti-inflammatory vs. pro-inflammatory effects of osteopontin and to clarify its impact on inflammation.

TNAP’s ectophosphatase activity is also involved in the modulation of TLR ligands, such as the double-stranded RNA mimic poly-inosine:cytosine (pI:C, TLR3 agonist) and microbial LPS (ligand of TLR4) [7]. Ligand dephosphorylation mitigates TLR-related stimulation of the inflammasome and cytokine secretion. Such effects appear to be clinically meaningful, e.g., in preterm neonates with late-onset sepsis [7,106]. The impact of TLR ligand processing on chronic inflammatory processes that trigger aging and related degenerative diseases, including Alzheimer’s disease, remains to be unraveled in more detail [107,108]. The role of TNAP in inflammatory processes in the intestine, especially in chronic inflammatory intestinal conditions like colitis, is of putative relevance, but the molecular mechanisms involved are largely unknown. In preclinical models of colitis, TNAP is characterized as a modulator of T-cell function [109]. However, often, the overlapping roles of TNAP and its intestinal isoenzyme IAP cannot clearly be separated in the intestine, although experiments in artificial systems document an unequivocal effect of TNAP on T-cell biology. In this context, another function of TNAP and its intestinal counterpart IAP has to be taken into account and this is dephosphorylation of microbial LPS, as already mentioned above (see Figure 2) [7]. This function is an ancient one that appears to be evolutionarily conserved in the phosphatase family and is important for the interface with the intestinal microbiome. As such, APs may have an anti-inflammatory effect by dephosphorylating the lipid A moiety of LPS, that is important for binding to the TLR receptor 4/MD-2 innate immune receptor complex and downstream pro-inflammatory signaling. Both IAP and TNAP are capable of catalyzing this reaction but their respective allotment in this performance in health and disease is hard to dissect in humans and mice [7,110].

TNAP’s ectonucleotidase activity controls the level of inflammation by converting the pro-inflammatory ATP to the anti-inflammatory nucleotide adenosine (see Figure 1 and Figure 2). High expression or overexpression of TNAP have documented anti-inflammatory effects via ATP degradation and production of adenosine, however, as an unwanted side effect, dystopic calcification can be stimulated, which can again trigger and support inflammatory calcifying conditions, such as calcifying tendinitis or inflammatory atherosclerotic plaques [111]. Generally, the balance between P1 and P2 receptor signaling is key for the net outcome of the level of inflammatory activity (see Figure 1).

Thus, talking about adenosine delivery by nucleotidases, one has to take into account that besides TNAP, there are several efficient membrane-associated nucleotidases contributing to the adenosine pool. At least four different sources contribute to the adenosine pool, (1) ATP dephosphorylation, (2) cAMP degradation, (3) nicotinamide adenine dinucleotide (NAD^+^)-dependent synthesis of cADPR (cyclic adenosine diphosphate ribose) and its degradation, and (4) adenosine release from the intracellular pool to the extracellular space via nucleoside transporters (only in neurons, see Figure 1).

Apart from TNAP, the enzymes ectonucleotide pyrophosphatases/phosphodiesterases 1–3 (CD203A–C, ENPP1–3), ectonucleoside triphosphate diphosphohydrolase 1 (CD39, ENTPDase1), and ecto-5′-nucleotidase (CD73, NT5E) are involved in the pre-receptor metabolism of nucleotides [112,113]. The stepwise dephosphorylation of ATP towards adenosine is mediated by TNAP, ENPP1, CD39, and CD73 (Figure 1) [112,113]. The second contribution to the adenosine pool comes from adenylate cyclase (AC) activation and the generation of intracellular cyclic AMP (cAMP), cAMP efflux through multidrug resistance protein 4 (ABCC4, MRP4), and the hydrolysis of released cAMP to AMP by ENPP2, followed by dephosphorylation to adenosine again by CD73 and putatively, TNAP [114]. The third contribution is based on a synthesis pathway, where cADPR, generated by the enzyme ecto-NAD-glycohydrolase (CD38, ADP-ribosyl cyclase 1) via NAD^+^, can be converted to AMP (by ENPP1) to then be dephosphorylated to adenosine by CD73 and again, putatively, TNAP [112,113]. Exclusively in the nervous system, adenosine can be released via nucleoside transporters into the extracellular space [115]. This, again with the exception of the neuronal system, renders CD73 a gatekeeper of adenosine generation. The question is, how much and under which conditions or tissue context does TNAP substantially contribute to this gatekeeper function (see Figure 1). In vitro enzymatic assays provide unequivocal evidence for efficient TNAP activity in the purinergic metabolic cascade from ATP to adenosine at any step [112,113]. However, its impact on the cellular microenvironment and finally on the in vivo situation in tissues may still be different and may vary between homeostatic and challenge situations [112,113].

Given its role as a dominant gatekeeper of the anti-inflammatory adenosine signaling system, CD73 is anti-nociceptive and protects against inflammatory damage, while also contributing to age-dependent decline in cortical plasticity [116]. CD73 preserves barrier function in multiple tissues, a role that is most evident in the respiratory system [116]. While the expression of CD73 on endothelia is documented across species, the expression of CD73 on immune cells is species-specific [117].

Adenosine downstream signaling is mediated via a set of P1 adenosine receptors (A1R, A2AR, A2BR, and A3R), which initiate G protein-coupled signaling and modulation of AC activity [97,118]. Adenosine receptors modulate cAMP production with differential effects, as A1R and A3R inhibit, whereas A2AR and A2BR enhance, cAMP production [95,119]. However, phosphorylation cascades downstream cAMP production may again converge into ERK1/2 activation and this may be stimulatory with all four receptors [95]. The biological effects of all receptors, with respect to cytokine release and inflammation, are uniformly anti-inflammatory, as it has been reported for the intestinal effects of adenosine signaling. The dark side of these effects, if chronically sustained, may be the support of immunosuppression and eventually the susceptibility to develop cancer [95]. Adenosine receptors are expressed in cells of the bone marrow and show effects on osteo- and adipogenesis [119]. Similar effects have been reported in other organs, such as liver and kidney [120,121]. Additionally, adenosine directly regulates cells of the immune system, such as myeloid cells [122].

Addressing the question whether TNAP contributes to the modulation of pro- vs. anti-inflammatory signaling via pre-receptor metabolism of ligands for P1 or P2 receptors under physiological or challenge conditions is a matter of partially controversial discussions. Some evidence that CD73 activity is sufficient to provide adequate adenosine levels comes from research addressing the ACDC syndrome (arterial calcification due to deficiency of CD73) [123]. This autosomal recessive disease is caused by loss-of-function mutations in the *CD73* gene and manifests as severe early atherosclerosis and calcifications in affected patients [123]. As a compensation for the prevalent CD73 deficit, TNAP was upregulated and produced adenosine simultaneously, supporting the calcification effects in mesenchymal stem cells from patients, but not from healthy controls [123]. In CD73 knockout mice, TNAP is upregulated and can compensate for the deficiency in adenosine production, although with an obvious difference between male vs. female animals [124].

In summary, TNAP can exert influence on inflammatory processes in variable ways, which are schematically depicted in Figure 2.

## 6. Conclusions

TNAP is an ancient, multi-systemically active enzyme with a broad number of substrates. Its activity is mainly composed of general ectophosphatase and specific ectonucleotidase components, where the latter, of course, also comprise dephosphorylation steps. It is involved in networks of enzymatic activities in physiology and pathology. Under certain conditions, TNAP function seems to be more or less redundantly backed up by enzymes with similar functions, but in case of bone mineralization, it appears to be one of two indispensable enzymes (TNAP and PHOSPHO1). Hence, the model disease Hypophosphatasia shows primarily a skeletal phenotype. This may have led to an unbalanced view on this enzyme in the past. More recently, the impact of TNAP on inflammatory activity is more and more apparent on multiple levels. TNAP and its substrates modulate a series of chronic inflammatory conditions, including aging-related degenerative diseases, such as atherosclerosis and Alzheimer’s disease, but also metabolic conditions, like insulin resistance and type 2 diabetes, occurring in the wake of chronic inflammation. Thus, based on the knowledge that has been accumulated about the role of TNAP in bone, it appears timely to widen the focus towards inflammation and metabolism in order to finally evaluate the therapeutic potential of agonists and antagonists in these complex systems.
